# Epidemiologic case investigation on the zoonotic transmission of *Staphylococcus* *aureus* infection from goat to veterinarians

**DOI:** 10.1111/zph.12836

**Published:** 2021-05-05

**Authors:** Silvia Piva, Jole Mariella, Monica Cricca, Federica Giacometti, Barbara Brunetti, Elisabetta Mondo, Lucia De Castelli, Angelo Romano, Irene Ferrero, Simone Ambretti, Mariana Roccaro, Giuseppe Merialdi, Alessandra Scagliarini, Andrea Serraino, Angelo Peli

**Affiliations:** ^1^ Department of Veterinary Medical Sciences University of Bologna Bologna Italy; ^2^ Department of Experimental, Diagnostic and Specialty Medicine Alma Mater Studiorum University of Bologna Bologna Italy; ^3^ Reference Laboratory for Coagulase‐Positive Staphylococci including Staphylococcus aureus Istituto Zooprofilattico Sperimentale del Piemonte Torino Italy; ^4^ Microbiology Department Policlinico Sant'Orsola Malpighi University of Bologna Bologna Italy; ^5^ Istituto Zooprofilattico Sperimentale della Lombardia e dell'Emilia–Romagna “Bruno Ubertini” Brescia Italy

**Keywords:** abortion, goat, professional zoonosis, *Staphylococcus aureus*, subtyping

## Abstract

*Staphylococcus aureus* infection led to a case of goat abortion, and four veterinarians contracted *S. aureus* infection from the goat during and after the abortion. Three veterinarians assisted a doe during the dystocic delivery of a dead foetus. Seventy‐two hours after the dystocia, which ended with the goat's death, the veterinarians who assisted during the kidding and the veterinarian who performed the necropsy showed the presence of multiple, isolated, painful pustules 1–5 mm in diameter located along their forearms and knees. *S. aureus* was isolated from the pustules of the veterinarians, the placenta and uterus of the goat, the organs (brain, thymus gland, abomasum, liver and spleen) of the foetus, the scrotum and eye swabs of the buck, and mammary pustules of another goat from the same herd. Histological analysis revealed purulent metritis and inflammation of the placental cotyledons. Additional investigations eliminated the chances of other infections. *S. aureus* isolates recovered from the veterinarians, goats, foetus and buck were sensitive to the tested anti‐microbials and did not encode staphylococcal enterotoxin genes (*sea*, *ser*, *sep*, *see*, *seg* and *sei*). The isolates were closely related, as indicated by the results of Fourier‐transform infrared spectroscopy and comparative whole‐genome sequencing analysis. The results of this study clearly support the hypothesis that an episode of professional zoonosis was caused by *S. aureus* infection during the abortion and also highlight the need for bacterial subtyping in epidemiological surveys.

**Impacts**

- The causative role of *Staphylococcus aureus* in a case of goat abortion was described
- The transmission of *S. aureus* from the goat to veterinarians evoked an episode of professional zoonosis
- This study suggests the potential use of FTIR in microbiological and outbreak investigations

## 1 INTRODUCTION

*Staphylococcus aureus* is a commensal organism and has long been recognized as an important pathogen in humans and in domestic and wild animals (Balasubramanian et al., 2017; Tong et al., 2015; Zhou et al., 2017). In humans, studies have shown nasal colonization by *S. aureus* in 30% of healthy carriers (van Belkum et al., 2009; Wertheim et al., 2005). *S. aureus* can cause various opportunistic infections (Tong et al., 2015), and some strains produce toxins that can lead to toxic shock syndrome or may be linked to staphylococcal food poisoning (Headrick et al., 1998). The risk of contact with methicillin‐resistant *S. aureus* is a major concern in nosocomial infections (Rubin et al., 1999), as it is associated with higher mortality rates and human health care costs (Köck et al., 2010).

In veterinary medicine, *S. aureus* is a common commensal bacterium and an important cause of abscesses, mastitis, pneumonia and meningitis (Balasubramanian et al., 2017; Zhou et al., 2017), causing significant economic losses in livestock production (Petersen et al., 2013; Verkade & Kluytmans, 2014). Dairy sheep and goat farms suffer significant economic losses due to staphylococcal infections in animals, with *S. aureus* being the primary causative agent of clinical mastitis in small ruminants (Bergonier et al., 2003).

*Staphylococcusaureus* isolates pose a potential public health risk, and the potential for the zoonotic transmission of staphylococci among livestock, companion animals and humans has been reported (Larsen et al., 2016; Pantosti, 2012).

Abortion, stillbirth and the delivery of weak lambs or kids are important issues in sheep and goat farming and have been associated with considerable economic losses. The cause can be determined in 47%–75% of caprine abortions (Chanton‐Greutmann et al., 2002). The major microbes associated with small ruminant abortion are *Chlamydophila abortus*, *Campylobacter fetus*, *Coxiella burnetii*, *Listeria monocytogenes*, *Toxoplasma gondii*, *Pestivirus D* (border disease virus) and *Aspergillus* spp., whereas *Escherichia coli*, *Salmonella* spp., *Bacillus* spp., *Staphylococcus* spp. and *Streptococcus* spp. are considered opportunistic pathogens.

Here, we describe a case of goat abortion caused by *S. aureus* infection and the transmission of *S. aureus* from the goat to three veterinarians who assisted the delivery and to the veterinarian who performed the necropsy procedure. This case highlights the need for accurate epidemiological surveillance.

## 2 MATERIALS AND METHODS

### 2.1 Case presentation and sample collection

**FIGURE 1:**
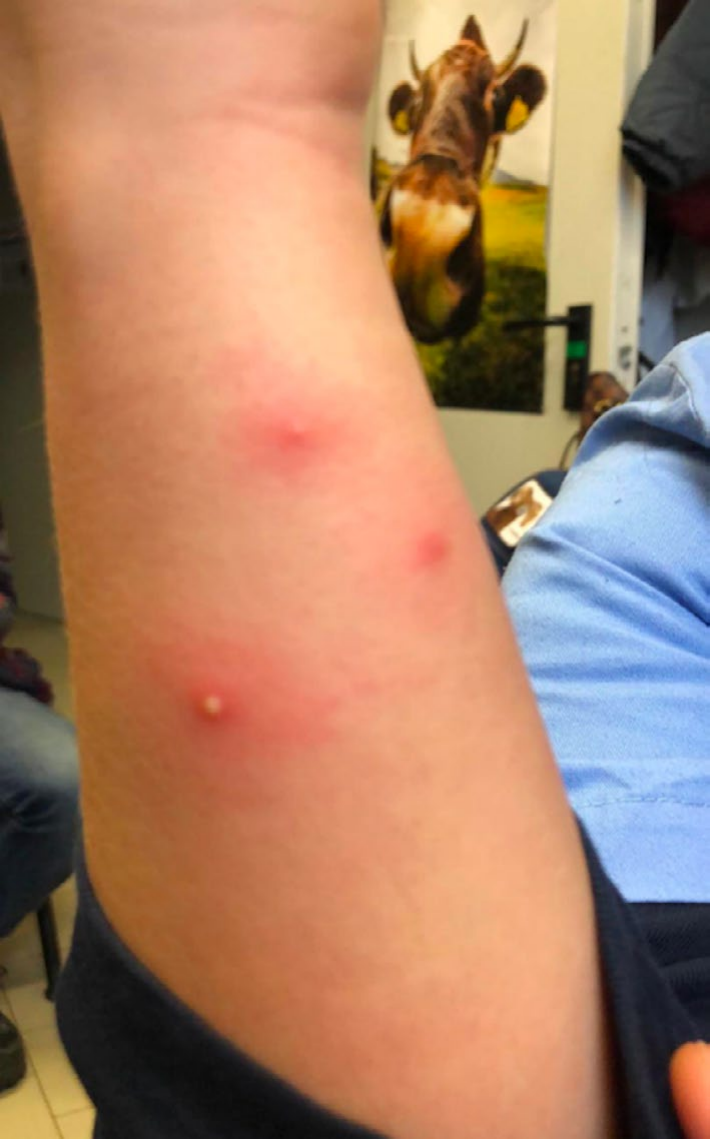
Picture of the pustules located along forearms

A goat, housed at the Department of Veterinary Medical Sciences of the University of Bologna, showed signs of parturition early in the morning; however, the amniotic sac or foetus was not visible at the vulvar lips. Foetal malpositioning, with the head of the foetus deviated downward and the limbs flexed, was observed. During the manual correction and traction of the foetus, performed by a veterinarians who wore scrubs, obstetric gloves and shoe covers (Vet 1), the goat began to exhibit signs of shock, such as cold extremities and bradycardia, and expired shortly thereafter. Two other veterinarians (Vet 2 and Vet 3), who assisted the obstetric veterinarian, also wore scrubs, gloves and the designated footwear. Necropsy revealed the presence of the foetus outside the uterus in a pathological position and a laceration of the uterus in the left horn, along with signs of bruising and disseminated intravascular coagulation in the lung. Vet 4 performed the necropsy wearing a gown, gloves and shoe covers. Seventy‐two hours later, the three veterinarians (Vet 1, Vet 2 and Vet 3) who assisted the kidding and the veterinarian (Vet 4) who performed the necropsy showed the presence of multiple, isolated, painful pustules of 1–5 mm in diameter along their forearms and knees. In particular, the pustules were located on the hands, forearms and knees of Vet 1, on the hands and forearms of Vets 2 and 3, and only on the forearms of Vet 4 (Figure 1). Vet 1 exhibited other clinical signs, including mild fever (37.3°C) that lasted for 1 day. No headache, myalgia or lymphadenopathy was observed in any of the veterinarians, and the skin lesions disappeared spontaneously within 3–5 days.

Based on these observations, a skin infection related to the goat abortion procedure was suspected. Samples were collected using sterile swabs from the intact pustules of the veterinarians, the placenta and uterus of the goat, and the foetal organs (brain, thymus gland, abomasum, liver and spleen) for bacteriological and histological examinations. From the goat herd, samples were collected from the scrotum and eyes of the buck and the mammary pustules of a secondary goat, and environmental samples were collected from the floor, gates of the boxes, water and milking machine for bacteriological examination.

### 2.2 Bacteriological and histological examinations and strain typing

All collected samples underwent routine aerobic and anaerobic bacterial cultures on blood agar, MacConkey agar and mannitol salt agar, purchased from Oxoid (United Kingdom) Presumptive colonies were submitted for preliminary biochemical tests, such as Gram staining and catalase and coagulase detection, and were definitively identified using MALDI‐TOF (Bruker Daltonics). In addition, sections from the placenta and uterus and the foetal abomasum and spleen were also processed for the isolation of *Arcobacter* spp., *Brucella* spp., *Listeria* spp., *C*. *fetus*, fungi and yeast as well as for the detection of *C*. *abortus*, *C*. *burnetii* and *Pestivirus D* (border disease virus). An anti‐microbial susceptibility test was performed on all *S. aureus* isolates using the disc diffusion method, according to the guidelines of the Clinical and Laboratory Standards Institute (CLSI) (Clinical & Laboratory Standards Institute, 2008), for amoxicillin/clavulanic acid, ampicillin, clindamycin, levofloxacin, oxacillin, tetracycline and trimethoprim/sulfamethoxazole.

Microorganisms were identified at the subspecies level using an IR Biotyper (Bruker Daltonics) using Fourier‐transform infrared spectroscopy (FTIR). The isolates were cultured on tryptose blood agar plates (Vakutainer Kima) for 24 hr at 37°C, and a loopful of bacterial cells was suspended in a Bruker suspension vial with inert metal cylinders after adding 50 μl of 70% ethanol. The suspensions were homogenized, and 15 μl of each suspension was dropped in triplicate on a silicon plate (Bruker Optics‐Daltonics) and dried.

### 2.3 Detection of staphylococcal enterotoxin genes and whole‐genome sequencing

Eight *S. aureus* isolates from the veterinarians (pustules of Vets 1 and 2), the goat (uterus and placenta), the foetus (abomasum and spleen) and the buck (eyes and scrotum) were used to detect staphylococcal enterotoxin genes (*sea*, *ser*, *sep*, *see*, *seg* and *sei*) using two multiplex PCR assays according to protocols of the European Union Reference Laboratory for Coagulase‐Positive staphylococci, including *S. aureus*.

Bacterial genomic DNA belonging to the eight *S. aureus* isolates was extracted using the QIAamp DNA Mini Kit (Qiagen). WGS was performed on the MiSeq platform (Illumina, San Diego) using paired‐end libraries that were prepared using the Nextera™ DNA Flex Library Prep Kit (Illumina), with a 150 bp read length. The WGS run had the following overall statistics: cluster passing filter of 99.36%, quality score ≥30 of 98.73% with a total yield of 11.57 Gbp achieved. The reads were first analysed using the Galaxy tool ‘FastQC Read Quality reports' accessed via the Galaxy public server at https://usegalaxy.org (Afgan et al., 2016) to provide quality control checks on raw sequence data. The raw reads were trimmed using the Galaxy tool Trimmomatic (Bolger et al., 2014) to trim the sequencing adapters and low‐quality regions, and the reads were assembled into genomes using Unicycler (ver. 0.4.1.1) in Galaxy (Wick et al., 2017). The assembled genomes were processed to determine the multilocus sequence typing (MLST) in silico using MLST 1.8 (accessed via https://cge.cbs.dtu.dk/services/MLST) (Larsen et al., 2012), to estimate the presence of potentially pathogenic genes using PathogenFinder 1.1 (accessed via https://cge.cbs.dtu.dk/services/PathogenFinder/) (Cosentino et al., 2013), to identify anti‐microbial resistance genes using KmerResistance 2.2 (accessed via https://cge.cbs.dtu.dk/services/KmerResistance/) (Clausen et al., 2016; International Working Group on the Classification of Staphylococcal Cassette Chromosome Elements, 2009), to identify the cassette chromosome *mec* to define methicillin resistance using SCCmecFinder 1.2 (accessed via https://cge.cbs.dtu.dk/services/SCCmecFinder/), and to detect virulence genes using VirulenceFinder 2.0 (accessed via https://cge.cbs.dtu.dk/services/VirulenceFinder//) (Joensen et al., 2014). The fastq files of the paired reads were processed using CSI Phylogeny 1.4 (accessed via https://cge.cbs.dtu.dk/services/CSIPhylogeny/) to call and filter single‐nucleotide polymorphisms (SNPs) and infer phylogeny based on the concatenated alignment of high‐quality SNPs (Kaas et al., 2014).

## 3 RESULTS

All specimens, excluding environmental specimens, exhibited monomicrobic growth of Gram‐positive, catalase‐positive, and coagulase‐positive cocci on blood agar and mannitol salt agar plates after 24 hr of incubation. One isolate from each sample was identified using MALDI‐TOF (Bruker Daltonics) with score values ranging from 2.09 to 2.18 for *S. aureus*. The anti‐microbial susceptibility test revealed that the isolates were susceptible to all tested anti‐microbials.

Additional investigations to exclude other infections revealed the presence of *Aspergillus* spp. only in the foetal abomasum.

**FIGURE 2:**
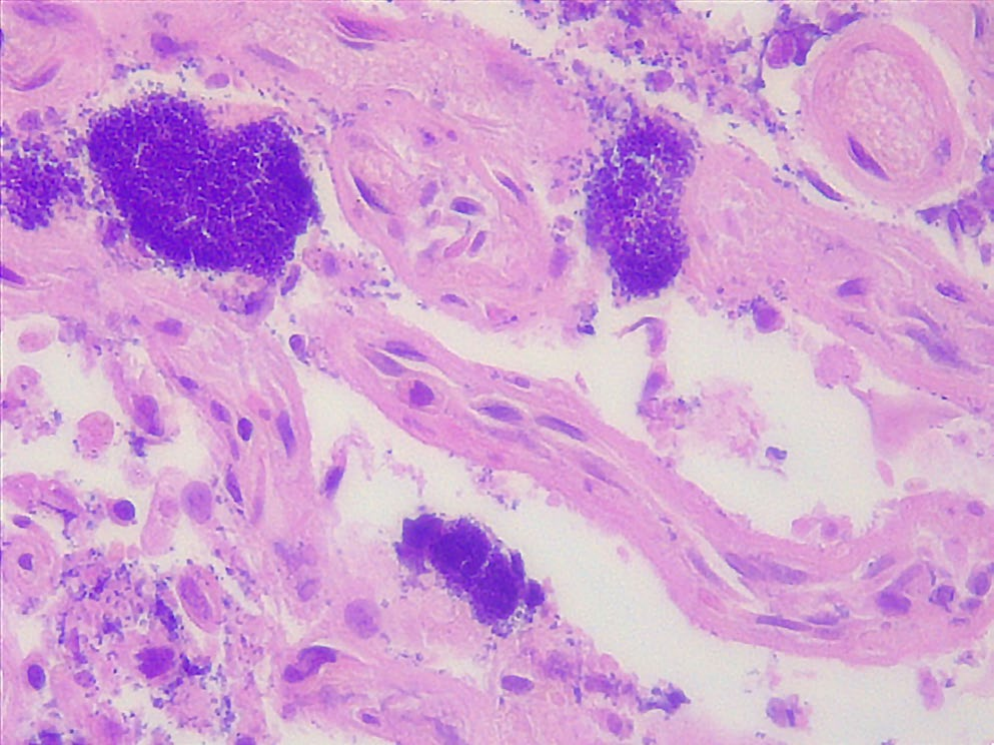
Apical area of a placentoma. This section shows multifocal large aggregates of basophilic extracellular bacteria. Several bacteria are also inside the cytoplasm of trophoblasts and endometrial epithelial cells; many of these cells are detached from the basal membrane of foetal villi and maternal stroma. Haematoxylin and eosin, 63X

Histological analysis revealed signs of purulent metritis and inflammation in the placental cotyledons. Numerous coccoid bacteria were observed in the samples collected from the placenta, uterus, and foetal organs, and purulent inflammation was observed in the uterine mucosa (Figure 2).

**FIGURE 3:**
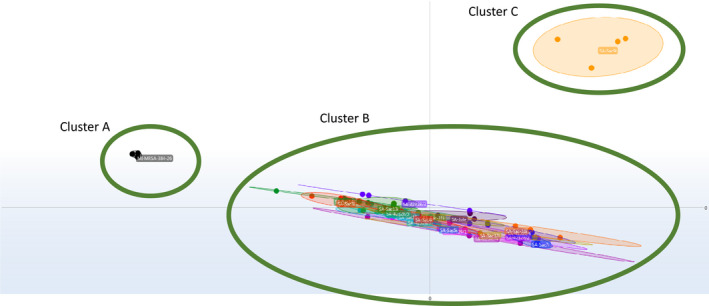
Scatter plot obtained by clustering IR spectra for *Staphylococcus aureus* (cut‐off, 0.185). IR spectra were grouped by the application of linear discriminant analysis (LDA). Cluster A: control *S. aureus* isolates; Cluster B: *S*. *aureus* isolated from humans, goat, foetus and buck; Cluster C: *S*. *aureus* isolated from the mammary pustules of a second different goat belonging to the same herd

Among the *S. aureus* isolates, two different clusters were identified using the IR Biotyper (Figure 3): the first included isolates from the veterinarians, goat, foetus, and buck, whereas the second included isolates from the mammary pustules of the secondary goat from the same herd.

All eight *S. aureus* isolates tested negative for the staphylococcal enterotoxin genes. The WGS data are presented in Table 1 In particular, in silico MLST indicated that all eight *S. aureus* isolates tested belonged to the strain ST700. This strain was isolated previously in Italy, principally from sheep, and was also detected in goats in a case linked to mastitis reported in 2009. Using PathogenFinder, a minimum of 295 to a maximum of 297 genes matched to the potential pathogenic families were retrieved. The results of KmerResistance 2.2 showed that none of the strains harboured anti‐microbial resistance genes, except the strain isolated from the eye swabs of the buck, which tested positive for the *tet*(k) gene. SCCmecFinder 1.2 predicted that all strains were methicillin‐sensitive *S. aureus*, as demonstrated by the disc diffusion and KmerResistance results. Analysis using VirulenceFinder 2.0 showed that the same pattern for virulence genes, including epidermal cell differentiation inhibitor B (*edin*B), gamma‐haemolysin chain II precursor A (*hlg*A), gamma‐haemolysin component B precursor (*hlg*B), gamma‐haemolysin component C (*hlg*C), leukocidin D component (*luk*D), leukocidin E component (*luk*E), aureolysin (*aur)*, serine protease A (*spl*A), serine protease B (*spl*B), and serine protease E (*spl*E), was observed in all samples.

**TABLE 1:** Whole‐genome sequencing data results

File	Isolation source	Maldi‐Tof	MLST	PathogenFinder	ResFinder	SCCmecFinder	VirulenceFinder
		Score		Probability of being a human pathogen			
37920–1	Human pustules_1	2.130	ST−700	0.982	Not found	MSSA	edinB, hlgA, hlgB, hlgC, lukD, lukE, aur, splA, splB, splE
37920–2	Human pustules 2	2.090	ST−700	0.982	Not found	MSSA	edinB, hlgA, hlgB, hlgC, lukD, lukE, aur, splA, splB, splE
37920–3	Buck's scrotum_	2.170	ST−700	0.981	Not found	MSSA	edinB, hlgA, hlgB, hlgC, lukD, lukE, aur, splA, splB, splE
37920–4	Buck's eyes	2.110	ST−700	0.981	tet (k)	MSSA	edinB, hlgA, hlgB, hlgC, lukD, lukE, aur, splA, splB, splE
37920–5	Goat's uterus	2.160	ST−700	0.982	Not found	MSSA	edinB, hlgA, hlgB, hlgC, lukD, lukE, aur, splA, splB, splE
37920–6	Placenta	2.160	ST−700	0.981	Not found	MSSA	edinB, hlgA, hlgB, hlgC, lukD, lukE, aur, splA, splB, splE
37920–7	Foetus’ abomasum	2.090	ST−700	0.982	Not found	MSSA	edinB, hlgA, hlgB, hlgC, lukD, lukE, aur, splA, splB, splE
37920–8	Foetus’ spleen	2.180	ST−700	0.982	Not found	MSSA	edinB, hlgA, hlgB, hlgC, lukD, lukE, aur, splA, splB, splE

**FIGURE 4:**
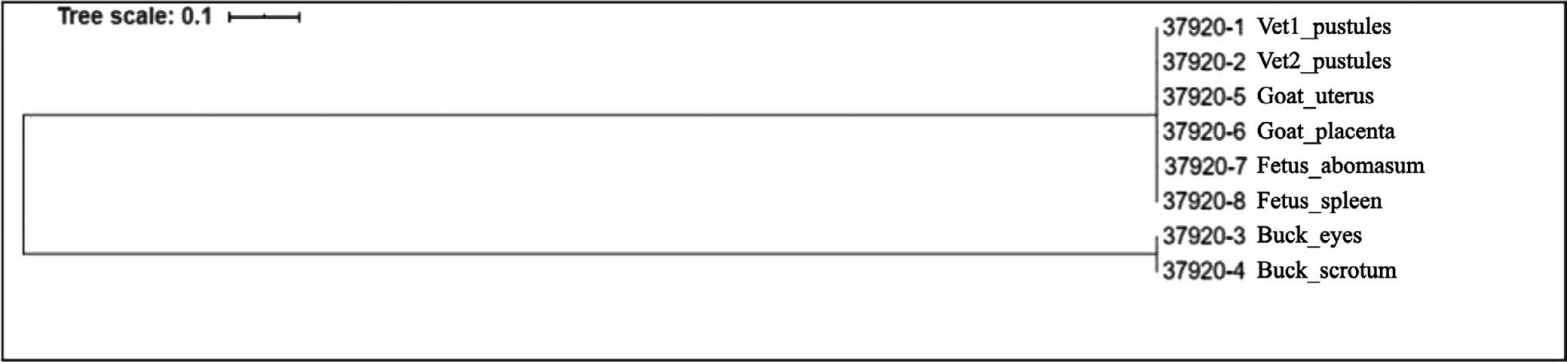
Single‐nucleotide polymorphism analysis of *Staphylococcus* *aureus* isolates from humans, goat, foetus and buck performed with CSI Phylogeny 1.4

The maximum likelihood tree obtained using SNP analysis with CSI Phylogeny 1.2 highlighted two distinct clusters: the first included *S. aureus* isolated from the veterinarians, the goat, and the foetus, and the second included *S. aureus* isolated from the buck (Figure 4).

## 4 DISCUSSION

The present study describes a case of abortion in a goat due to *S. aure*us infection and the zoonotic transmission of *S. aureus* from the goat to four veterinarians. To our knowledge, this is the first study to show the zoonotic transmission of *S. aureus* from a goat to veterinarians.

This study highlights the need for accurate epidemiological surveillance for delineating outbreaks and the source(s) of infection.

Bacterial strain typing can aid in outbreak investigation by revealing clonality, identifying the potential routes of transmission or source attribution, and linking clinical isolates to environmental reservoirs. In cases of *S. aureus* infection, subtyping is commonly performed using pulsed‐field gel electrophoresis, spa typing, and MLST, although each of these methods has certain limitations. In this study, FTIR was used for *S. aureus* subtyping, and the results were subsequently compared with the WGS results, given that WGS provides information on the genome structure and gene content, which is largely applied in outbreak investigations and genomic comparison (Macori et al., 2020). FTIR is a spectrum‐based technique that quantifies the absorption of infrared light by molecules present in bacterial cells. The generated infrared spectra provide a fingerprint of the composition of nucleic acids, proteins, lipids, and carbohydrates in cells. Therefore, each microorganism has a highly specific infrared absorption signature correlated with genetic information, which facilitates microorganism identification at the subspecies level (Rakovitsky et al., 2020; Venning & Scherer, 2013). FTIR is a promising tool for rapid discrimination among strains in outbreak investigations (Johler et al., 2013) as well as in source attribution studies (Harmsen et al., 2003). In this study, analyses of the FTIR and WGS results showed that the *S. aureus* isolates obtained from the veterinarians, goat, foetus, and buck were closely related, suggesting that the buck was the source of infection; this led us to confirm this as a case of professional zoonosis. The *S. aureus* isolates appeared to be particularly non‐virulent, showing the same unremarkable pattern of virulence genes; the isolates were sensitive to all the tested anti‐microbials, and no staphylococcal enterotoxin genes were detected using PCR. Based on these findings, the transmission of *S. aureus* from the goat to the veterinarians was considered to result from a high infectious load in the goat, which possibly led to foetal death and the zoonotic event.

The veterinarians may have come in contact with the pathogen through contact between the skin and the outer surface of the long‐sleeve obstetric gloves when they were removed or via perforations in the gloves. Moreover, both the scrubs and gowns used by the veterinarians were permeable. In cases where splashing or soaking with potentially infectious liquids is anticipated, the use of impermeable outerwear, such as plastic aprons or coveralls, is recommended (Elchos et al., 2008).

The results obtained using FTIR and WGS analyses were comparable in terms of their ability to link different isolates for epidemiological purposes. WGS provides extensive information, such as evaluation of the presence of virulence factors or mobile genetic elements, but remains unsuitable for routine use owing to its high cost and the complexity of the analytical process. In comparison, FTIR is a rapid and inexpensive method that can be useful for outbreak investigation, even though it provides less information than WGS. The results of this study support the use of FTIR for the short‐term epidemiological investigation on source attribution in *S. aureus* infection, and even though the findings from this case study require further investigation, the use of FTIR is strongly recommended for countering the general opinion that this technique is underused in microbiological studies and its potential in routine microbiology is underestimated (Novais et al., 2019).

In conclusion, this study reports a case of zoonosis and highlights the necessity of subtyping analysis for epidemiological surveillance and source attribution. Two different methods, with different and complementary characteristics, were used, and both demonstrated the correlation among isolates from different sources and traced the origin of the infection. FTIR proved to be an efficient, rapid, and inexpensive tool for epidemiological investigation on source attribution, whereas WGS, which required more time and was more complex than FTIR, facilitated the detailed characterization of the *S. aureus* isolates.

## CONFLICT OF INTEREST

None.

## AUTHOR CONTRIBUTIONS

GM, SA, EM, AS and FG dealt with microbiological aspects, while AP, MR,CP and JM dealt with clinical aspects. BB dealt with histological analysis, AR and FI performed the library preparation and sequencing and bioinformatics analysis of WGS data; SP, AS and FG wrote the manuscript; LD proofread the manuscript and validate the data. MC performed FTIR analysis. All authors read and approved the final manuscript.

## ETHICS APPROVAL AND CONSENT TO PARTICIPATE

We do not have an ethical statement because all the information were collected during the routine diagnostic procedures. The owner was informed about data publication.

## CONSENT FOR PUBLICATION

Authors have the written consent of goat owner, namely the person in charge of the goat's teaching herd of the Clinic for Ruminants of the Department of Veterinary Medical Sciences, University of Bologna for publication of personal and clinical details in this study. All veterinarians involved in the manuscript are Authors giving their consent for personal or clinical details.

## Data Availability

The datasets used and/or analysed during the current study are available from the corresponding author on reasonable request.
